# *In vivo* interactions of continuous flucloxacillin infusion and high-dose oral rifampicin in the serum of 15 patients with bone and soft tissue infections due to *Staphylococcus aureus* - a methodological and pilot study

**DOI:** 10.1186/2193-1801-3-287

**Published:** 2014-06-07

**Authors:** Christian Garzoni, Ilker Uçkay, Wilson Belaieff, Dominique Breilh, Domizio Suvà, Elzbieta Huggler, Daniel Lew, Pierre Hoffmeyer, Louis Bernard

**Affiliations:** Department of Infectious Diseases, Geneva University Hospitals and Medical School, 4 Rue Gabrielle Perret-Gentil, 1211 Geneva, Switzerland; Clinic for Infectious Diseases, University of Berne, Inselspital, Bern Switzerland; Orthopaedic Surgery Department, Geneva University Hospitals and Medical School, 4 Rue Gabrielle Perret-Gentil, 1211 Geneva, Switzerland; Departments of Pharmacokinetics and Clinical Pharmacy, Victor-Segalen University, Bordeaux, France; Department of Infectious Diseases, Tours University Hospital, Tours, France

**Keywords:** Flucloxacillin, Rifampicin, Synergism, Pharmacodynamics, *Staphylococcus aureus*

## Abstract

**Background:**

Increased antibiotic resistance against *Staphylococcus aureus* and low penetration into bone requires regimen optimization of available drugs.

**Methods:**

We evaluate pharmoacokinetic and pharmacodynamic parameters (PK/PD) as well as *in vivo* interactions of continuous flucloxacillin 12 g/d administration combined with high dose oral rifampicin 600 mg bid in the serum of 15 adult patients with bone and soft tissue infections. We use the patient’s own serum directed against his own isolated *S. aureus* strain to reproduce *in vivo* conditions as closely as possible.

**Results:**

The continuous flucloxacillin infusion constantly generated plasma free drug levels largely exceeding the serum minimal inhibitory concentrations (mean 74-fold). Combination with rifampicin significantly increased flucloxacillin levels by 44.5%. Such an increase following rifampicin introduction was documented in 10/15 patients, whereas a decrease was observed in 1/15 patients. Finally, all infections were cured and the combination was well tolerated.

**Conclusions:**

In this *in vivo* methodological pilot study among adult patients with orthopaedic infections due to *S. aureus*, we describe a new method and reveal substantial but inconsistent interactions between flucloxacillin and rifampicin, of which the clinical significance remains unclear.

## Introduction

*Staphylococcus aureus* is one of the most prevalent pathogen in bone and soft-tissue infections associated with and without foreign material. When implants are involved, *S. aureus* develops a biofilm in which most antimicrobial agents reveal inability to kill non-growing bacteria. The only clinically available exception is rifampicin/rifampin (Uçkay et al. 2009). Since rifampicin monotherapy may lead to rapid emergence of rifampicin-resistant *S. aureus*, this drug should always be combined with other systemic antibiotics (Widmer et al. 1992; Zavasky and Sande 1998).

Continuous intravenous administration of a β-lactam antibiotics has been suggested more beneficial than intermittent drug use (Boselli et al. 2008; Drusano 2004; Landersdorfer et al. 2007). Flucloxacillin is a synthetic penicillinase-resistant penicillin similar to oxacillin or methicillin; with excellent anti-staphylococcal activity. For severe bone and soft tissue infections due to *S. aureus*, whether associated with orthopaedic implants or not, only limited data is currently available for flucloxacillin in continuous infusion alone (Howden and Richards 2001; Hackbarth et al. 1986; Leder et al. 1999) or in combination with rifampicin. Furthermore, development of new antimicrobial drugs against *S. aureus* by the industry is limited (Jugun et al. 2013). Therefore, new pharmacokinetic and pharmacodynamic (PK/PD) studies *in vivo* are required to optimize the potency of today’s available antimicrobial regimens (Le and Bayer 2003; Czekaj et al. 2011). In this methodological and pilot study, we prospectively investigate activity parameters of a combined oral rifampicin and continuous flucloxacillin regimen in 15 adult hospitalized patients. Methodologically speaking, we use the patient’s own serum directed against his own isolated *S. aureus* strain to reproduce *in vivo* conditions as closely as possible. This method may be clinically more relevant than *in vitro* studies on synergism/antagonism. Of note, this study wasn’t designed to measure clinical outcome, and is focused on pharmocologic parameters *in vivo* only.

## Methods

### Subjects and treatment

Fifteen adult patients hospitalized for acute bone and soft tissue infections due to *S. aureus* were prospectively included in this methodological pilot study. Informed patient consent was obtained, in accordance with local institution policy. All subjects had microbiological identification of *S. aureus* in more than two concordant intraoperative bacterial cultures and a normal hepatic and renal function (creatinin-clearance > 50 ml/min). Exclusion criteria were polymicrobial infections, co-medication with any drug known for significant interaction with rifampicin, patients with allergy or intolerance to flucloxacillin and/or rifampicin, and hepatitis (serum transaminase levels >3× upper normal limit or cirrhosis CHILD B or C).

All patients were antibiotic-naïve prior to flucloxacillin/rifampicin therapy being started. Continuous intravenous flucloxacillin 12 g/d without loading dose was initiated as soon as *S. aureus* infection was suspected on the basis of a positive Gram-staining while awaiting culture pathogen identification. After 72 h, flucloxacillin was considered at steady state, because of its serum half-life of 0.75-1.5 h. Oral rifampicin 600 mg bid was added after culture confirmation and kept throughout the therapy. Duration of combined treatment with flucloxacillin and rifampicin followed current recommendations (Trampuz and Zimmerli 2006; Bernard et al. 2010). Surgery was performed according to standard of care. Staphylococci species were characterized by slidex agglutination (Pastorex®, BIO-RAD) and the ID32 Staphylococcus Gallery (bioMérieux, Marcy L’Etoile, France) and according to Clinical and Laboratory Standard Institute guidelines (CLSI 2008).

### Pharmacologic analyses

Serum was collected at three predetermined time points. A: 72 h after initiation of flucloxacillin; B: at 48 h after rifampicin treatment onset but 30 minutes before the fifth dose was delivered (rifampicin trough level); C: 1 hour after fifth rifampicin dose (rifampicin peak level). At each time point, serum flucloxacillin (A,B,C) and rifampicin (B,C) concentrations were assessed using high liquid pressure chromatography technique. A specific and automatable assay was developed for simultaneous flucloxacillin and rifampicin quantification in human plasma. A Prontosil C18 AQ + 150 × 4.6 mm column was used for chromatographic separation. Voriconazole was used as internal standard (IS). UV detection was set at three wavelengths: 220 nm for flucloxacillin, 340 nm for rifampicin and 260 nm for IS. Retention times were 3.8 min, 6 min. and 7 min. for flucloxacillin, rifampicin and IS respectively. Rifampicin and flucloxacillin were extracted from plasma and mixed with IS solution, an orthoboric acid pH 3.5-solution and an ascorbic acid solution to prevent rifampicin oxidation. Limits of quantification and detection were 0.25-0.1 μg/ml for flucloxacillin, and 0.1-0.03 μg/ml for rifampicin, respectively. Data’s validation for accuracy in intra and inter-day precision were good. Coefficient of variation for flucloxacillin was between 3.43% and 8.58% and accuracy between 94.2% and 103.7%. For rifampicin, the corresponding numbers were between 1.07% and 7.54% and between 96.0% and 106.4%, respectively.

Flucloxacillin steady-state concentrations were measured before 600 mg rifampicin was initiated (time point A) and at two time points afterwards (B and C). The observed minimum and maximum concentrations of rifampicin were assumed to be at time point B (trough level) and C (peak level 1 h after), respectively. Steady-state concentration-time data for flucloxacillin were analyzed with a standard pharmacokinetic method by using Kinetica™ software (Version 4.4, San Diego, USA). Steady-state concentration-time data for rifampicin were analyzed by population pharmacokinetic methods by using NPEM™ software (Los Angeles, USA).

### Serum minimal inhibitory concentration (SMIC) and bactericidal concentration (SMBC)

For every patient, SMIC was determined at every time point using the microdilution method. Briefly, patient’s serum was incubated for 24 h at 37°C after being added 10E^6^ colony forming units of *S. aureus* per serum milliliter. The *S. aureus* used was the same isolate that was isolated from the patient in question. SMIC was defined as the highest dilution that did not exhibit visible growth. Tests were performed in accordance with National Committee for Clinical Laboratory Standards recommendations (NCCLS 1999). The SMBC was defined as the concentration at which growth plates displayed killing ≥ 99.9% of the inoculum. All experiments for SMIC and SMBC were conducted in triplicate.

### Statistical analysis

Group comparisons were performed using the Wilcoxon-ranksum-test or the Kruskal-Wallis-test, as appropriate. *P* values ≤0.05 (two-tailed) were significant. Analyses were performed with STATA™ software, 9.0 (Corporate Station, USA).

## Results

### Patients and outcome

A total of 15 patients (age, 35-75 years) with *S. aureus* infections were enrolled according to inclusion/exclusion criteria: 4 cellulitis with abscesses, 4 native joint infections, 3 infected orthopaedic implants, 2 spondylodiscitis, 2 bursitis and 1 case of pin-track infection with acute osteomyelitis. One patient had both cellulitis and osteomyelitis. Two episodes were bacteremic. Surgery was performed in all patients: drainage (8*×*), debridement (3*×*), bursectomy (2*×*), one-stage prosthesis exchange (1*×*) and pin removal (1*×*). All subjects completed the study and had complete infectious remission after the completion of therapy with a minimum follow-up of 12 months. Duration of treatment was variable according to the infection (14 to 41d, mean 23 days). All *S. aureus* strains were highly sensitive to both studied drugs. SMIC for flucloxacillin ranged between 0.25-1 μg/ml (mean 0.78 μg/ml), and SMIC for rifampicin ranged between 0.015-0.03 μg/ml (mean 0.019 μg/ml) (CLSI 2008). Continuous flucloxacillin generated a mean serum level of 34 μg/ml (range 19.9-65.3). After adding rifampicin, on average flucloxacillin concentration increased significantly by 44.6% to a mean serum value of 45.6 μg/ml (range, 28.7-65.6) (*p* = 0.0008). In contrast, a highly significant difference was documented between SMIC at time point A and C, with a mean value increasing from 1:130 to 1:412 (*p* < 0.001). Only one patient showed decreased activity after adding rifampicin (Table 1). There was no occurrence of significant medicamentous hepatitis with serum transaminases levels above the upper limit or liver insufficiency.

**Table 1:** **Reciprocal serum minimal inhibitory and bactericidal dilutions** ^**+**^

	Reciprocal serum minimal inhibitory dilutions (reciprocal titer)^+^				Reciprocal serum minimal bacterial dilutions (reciprocal titer)^+^			
	FOXA alone	FOXA and RIF			FOXA alone	FOXA and RIF		
				Activity change after RIF combination (*)				Activity change after RIF combination (*)
Patient	TP-A	TP-B	TP-C		TP-A	TP-B	TP-C	
1	32	128	256	2.5	8	<2	<2	-3
2	256	64	256	-1	<2	4	<2	1
3	32	64	1024	3	<2	<2	1024	9
4	128	1024	1024	3	<2	32	32	5
5	128	128	128	0	<2	<2	16	2
6	512	64	128	-2.5	<2	<2	<2	0
7	32	128	32	1	4	<2	<2	-2
8	128	512	512	2	2	<2	<2	-1
9	64	256	512	2.5	64	<2	<2	-6
10	32	128	256	2.5	8	<2	<2	-3
11	128	512	1024	2.5	128	512	1024	2.5
12	64	512	128	2	4	<2	<2	-2
13	128	512	512	2	2	<2	2	-0.5
14	32	128	128	2	16	<2	<2	-4
15	256	128	256	-0.5	64	<2	<2	-6

FOXA = Continuous intravenous flucloxacillin 12 g/d. RIF = Oral rifampin 600 mg bid.

^+^Units are not written to spare space. Units are in μg/ml.

*Activity change expressed in dilutions. Calculated as difference between activity with FOXA alone (time point A) and the mean activity in the 2 conditions measured after RIF adjunction (time points B and C). Positive values denote increase activity, negative ones decreased activity.

## Discussion

We performed a methodological pilot study using the patient’s own serum directed against his own isolated *S. aureus* strain to reproduce *in vivo* conditions as closely as possible. We furthermore demonstrate significant pharmacologic interaction during simultaneous administration of continuous 12 g per day flucloxacillin and oral rifampicin 600 mg twice daily in 15 adult patients with various musculoskeletal infections due to *S. aureus.* Of note, our study did not demonstrate true synergism when using this combined regimen. The titer increase was only 3-fold when two antibiotic treatments were administered simultaneously in comparison to one drug alone. There was a wide discrepancy between inhibitory and bactericidal serum dilutions in the majority of patients. This finding is inconsistent with what would be expected for bactericidal drugs in which the SMBC is usually within 4-fold of the SMIC.

Literature is controversial regarding synergism vs. antagonism of flucloxacillin combination with rifampicin/rifampin (Brandt et al. 1994; Hackbarth et al. 1986; Massanari and Donta 1978; Swanberg and Tuazon 1984; Van der Auwera and Klastersky 1983; Van der Auwera et al. 1983, 1985; Zak et al. 1983; Zinner et al. 1981a, [b]). Overall, results between different *in vitro* studies correlate poorly and no single method has currently been proven superior to another (Bliziotis et al. 2007; Perlroth et al. 2008). Therefore, we have opted to study pharmagologic activities using the patient’s own serum directed against his own isolated *S. aureus* strain to reproduce *in vivo* conditions as closely as possible. Thus, our method of testing patient’s own serum with patient’s own *S. aureus* is novel to the best of our knowledge. Most of our patients’ serum collected under combined therapy showed enhanced antistaphylococcal flucloxacillin activity when tested *in vitro*. Sera collected at peak rifampicin concentration showed an increased, unchanged or decreased effect in 10 (67%), 4 (27%) and 1 (7%) cases respectively, when compared with sera collected before rifampicin introduction.

In conclusion, we report that in continuous infusion with 12 g/d, serum flucloxacillin levels constantly exceeded by several folds the MIC for *S. aureus* and the association with rifampicin may further increase these serum levels. We also demonstrate that combination of rifampicin with a flucloxacillin may partially lead to an increased bactericidal effect, which was, however, not consistent in all patients. Our study used the patient’s own serum directed against his own isolated *S. aureus* strain to reproduce *in vivo* conditions as closely as possible. The method used in the present study may be clinically more relevant than *in vitro* studies on synergism/antagonism. This was a methodological, pilot, and an *in vivo* pharmocologic study and not a clinical outcome study. All 15 patients were considered cured after a minimal follow-up of 12 months; however, our small cohort of patients makes it impossible to assess the clinical efficacy and relevance of our flucloxacillin and rifampicin serum interaction findings. Treatment may as well have been successful with a combination of surgery and flucloxacillin alone, particularly in the case of soft tissue infections such as cellulitis and bursitis.
